# Exercise-related hemoconcentration and hemodilution in hydrated and dehydrated athletes: An observational study of the Hungarian canoeists

**DOI:** 10.1371/journal.pone.0277978

**Published:** 2022-12-30

**Authors:** Zsolt Komka, Brigitta Szilágyi, Dóra Molnár, Bence Sipos, Miklós Tóth, Balázs Sonkodi, Pongrác Ács, János Elek, Máté Szász

**Affiliations:** 1 Department of Health Sciences and Sports Medicine, Hungarian University of Sports Science, Budapest, Hungary; 2 Heart and Vascular Center, Semmelweis University, Budapest, Hungary; 3 Hungarian Canoe Federation, Budapest, Hungary; 4 Institute of Mathematics, Budapest University of Technology and Economics, Budapest, Hungary; 5 Institute of Mathematics and Statistical Modelling, Corvinus University of Budapest, Budapest, Hungary; 6 Faculty of Natural Sciences Department of Geometry, Budapest University of Technology and Economics, Budapest, Hungary; 7 Faculty of Health Sciences, University of Pécs, Pécs, Hungary; 8 Department of Laboratory Medicine, Semmelweis University, Budapest, Hungary; 9 Szentágothai Research Center, Pécs, Hungary; 10 Department of Inorganic and Analytical Chemistry, University of Debrecen, Debrecen, Hungary; 11 Synlab Hungary Ltd., Budapest, Hungary; Poznan University of Physical Education, POLAND

## Abstract

Hemoconcentration during exercise is a well-known phenomenon, however, the extent to which dehydration is involved is unclear. In our study, the effect of dehydration on exercise-induced hemoconcentration was examined in 12 elite Hungarian kayak-canoe athletes. The changes of blood markers were examined during acute maximal workload in hydrated and dehydrated states. Dehydration was achieved by exercise, during a 120-minute extensive-aerobic preload. Our research is one of the first studies in which the changes in blood components were examined with a higher time resolution and a wider range of the measured parameters. Hydration status had no effect on the dynamics of hemoconcentration during both the hydrated (HS) and dehydrated (DHS) load, although lower maximal power output were measured after the 120-minute preload [HS Hemoglobin(Hgb)_Max_ median 17.4 (q1 17.03; q3 17.9) g/dl vs. DHS Hgb_Max_ median 16.9 (q1 16.43; q3 17.6) g/dl (n.s); HS Hematocrit(Hct)_Max_ 53.50 (q1 52.28; q3 54.8) % vs. DHS Hct_Max_ 51.90 (q1 50.35; q3 53.93) % (n.s)]. Thirty minutes after the maximal loading, complete hemodilution was confirmed in both exercises. Dehydration had no effect on hemoconcentration or hemodilution in the recovery period [HS Hgb_R30’_ 15.7 (q1 15.15; q3 16.05) g/dl (n.s.) vs. DHS Hgb_R30’_ 15.75 (q1 15.48; q3 16.13) g/dl (n.s.), HS Hct_R30’_ 48.15 (q1 46.5; q3 49.2) % vs. DHS Hct_R30’_ 48.25 (q1 47.48; q3 49.45) % (n.s.)], however, plasma osmolality did not follow a corresponding decrease in hemoglobin and hematocrit in the dehydrated group. Based on our data, metabolic products (glucose, lactate, sodium, potassium, chloride, bicarbonate ion, blood urea nitrogen) induced osmolality may not play a major role in the regulation of hemoconcentration and post-exercise hemodilution. From our results, we can conclude that hemoconcentration depends mainly on the intensity of the exercise.

## Introduction

During short-term maximal and prolonged submaximal exercises, both blood composition and fluid spaces can change dynamically, but their regulation is not sufficiently clarified; however, these changes basically determine hemodynamics and physical performance.

It is a well-known phenomenon that intense exercise can cause hemoconcentration. Because physical exertion causes many physiological changes in the body, several theories explain the phenomenon, but the process is likely to be complex or multifactorial. The intramuscular concentration of metabolites increases during exercise; their accumulation can enhance the osmotic gradient [1]. This, beside the sympathetic autonomic nervous system activity [2] and increased arterial pressure [3] results in the filtration of plasma into the interstitial space [4]. In addition to the plasma shift mechanism, adrenergic contraction of the spleen during intense exercise, delivering erythrocytes concentrate into the blood, may also contribute to hemoconcentration [5].

Increased erythrocytes count through hemoconcentration can locally reduce peripheral vascular resistance by directly increasing endothelial vasodilating NO synthesis [6] as well as ATP, which can also stimulate NO formation [7]. Beside the (⍺1) vasoactive adrenergic regulation, both processes result in dilatation of the precapillary resistance arterioles, thereby increasing local (muscular) blood flow. As a result of physiological hemoconcentration, the hemoglobin level increases during intense exercise, beneficially improving the O_2_ supply to muscle cells, allowing the muscle to function more economically. In the euhydrated state, physiological hemoconcentration can increase performance, and fluid replacement can even impair performance during short-term high intense exercise [8].

However, during long-term exercise or training in extremely hot and/or humid environments–with inadequate fluid replacement–the intravascular hemoconcentration continues to increase. Due to dehydration, the adaptive effects of hemoconcentration gradually disappear, performance decreases [8], or in some cases, health damage or a dangerous condition might develop [9–13].

A loss of total body water equivalent to or greater than 2% of body weight can significantly reduce performance at prolonged submaximal exertion [9, 10] and cause a decrease in muscle strength and function [14, 15]. The exact process of dehydration is unknown, and with the progression of dehydration, the rearrangement rate of fluid spaces is not sufficiently clarified. During dehydration due to external heat, in an animal model, approx. at 10% total body weight loss, 40% of the decrease in total body water content was attributed to muscle fluid loss. During thermal dehydration, this redistribution of water across the muscle cell membrane depends primarily on the osmotic gradient [9] and the activity of ion pumps [16]. However, when this external thermal effect is combined with exercise (extrinsic and intrinsic factors), metabolic changes due to muscle activity can modify water movement from skeletal muscle tissue.

From a practical point of view, it is important how long the hemoconcentration phenomenon lasts physiologically and how long it is beneficial for sports performance.

Several research groups have shown that the concentrated hemoglobin and hematocrit return to resting levels 30 minutes after a single acute anaerobic exercise [17–19]. Exercise-induced hemoconcentration was also described after high-intensity interval training (HIIT) [20]. Hemoglobin and hematocrit parameters increased immediately after HIIT and their values began to return to resting levels 3 h after exercise, and completely returned to resting levels 6 h after exercise, moreover overcompensation can occur [20]. Our previous study described that in the hydrated state, hemoconcentration also develops under the effect of an incremental dynamic maximal loading. This physiological response is a temporary, short-term process, with hemodilution occurring in 7–10 minutes post-exercise [19].

The aim of our study was to better understand the changes in fluid spaces and blood components in hydrated and dehydrated states in athletes under dynamic (cyclic type) loading. Our goal was to determine the extent to which dehydration affects the degree of hemoconcentration and to investigate the extent of hemodilution following maximal exercise during hydrated and dehydrated conditions.

## Methods

### Ethical statement

Our study was performed in accordance with the Declaration of Helsinki of the World Health Association, with the approval of the Scientific and Research Ethics Committee of the Hungarian University of Sports Science (TE-KEB/No8/2019.). All subjects provided written informed consent to the study.

### Study design

Our study was a self-controlled observational trial. The study procedure was conducted during the regular canoe sprint competition period of the 2019 season between May and August.

### Subjects

In our study, twelve elite Hungarian (Caucasian race) male kayak-canoe athletes (11 kayakers and one canoeist, median age 18.0 (q1 18.0; q3 21.3) years, training volume 16.5 (q1 15.75; q3 18.50) hours/week) were examined twice with a difference of 1 week. The workload of the study requires advanced aerobic endurance, so untrained volunteers could not be selected as a control group. Subjects with any symptomatic illnesses or receiving any medication were excluded from our study.

Study subjects were instructed not to consume alcohol or caffeinated beverages for 24 hours prior to the procedures. The day before the study, a standard carbohydrate (55%), fat (30%), protein intake (15%), and at least 3000ml of fluid (or the usual amount of fluid consumed by the athlete) were prescribed to the volunteers by a registered dietitian. One hour before the test, 300 ml of water was given, and then no liquid intake or food consumption was allowed until the end of the procedure.

### Methodology

The studies were performed in the morning (between 8–12 a.m.), ensuring the properly regenerated condition of the athletes. Before the study protocol, morning training was not allowed. The experiments were performed under air-conditioned laboratory conditions at a temperature of 22–23°C and a humidity of 30–40% at an altitude of 145m above sea level.

#### Short protocol

On the first test day (short protocol, hydrated state, HS), an exercise ramp protocol was performed with a Schiller ERG 911 recumbent bicycle ergometer at a constant pedal speed of 70-80/min with a 50Watt power increase every 3 minutes until maximum exhaustion (vita maxima type protocol). Samples and data were collected at rest before exercise; in the aerobic range (controlled by respiratory exchange ratio (RER = 0.9)), on the anaerobic threshold (RER = 1.0); at a maximum load; and at the 5th and 30th minutes of the recovery period. The load was continuous during sampling and was not interrupted for the duration of blood taking. Blood samplings lasted an average of 15–20 seconds.

#### Long protocol

**Fig 1:**
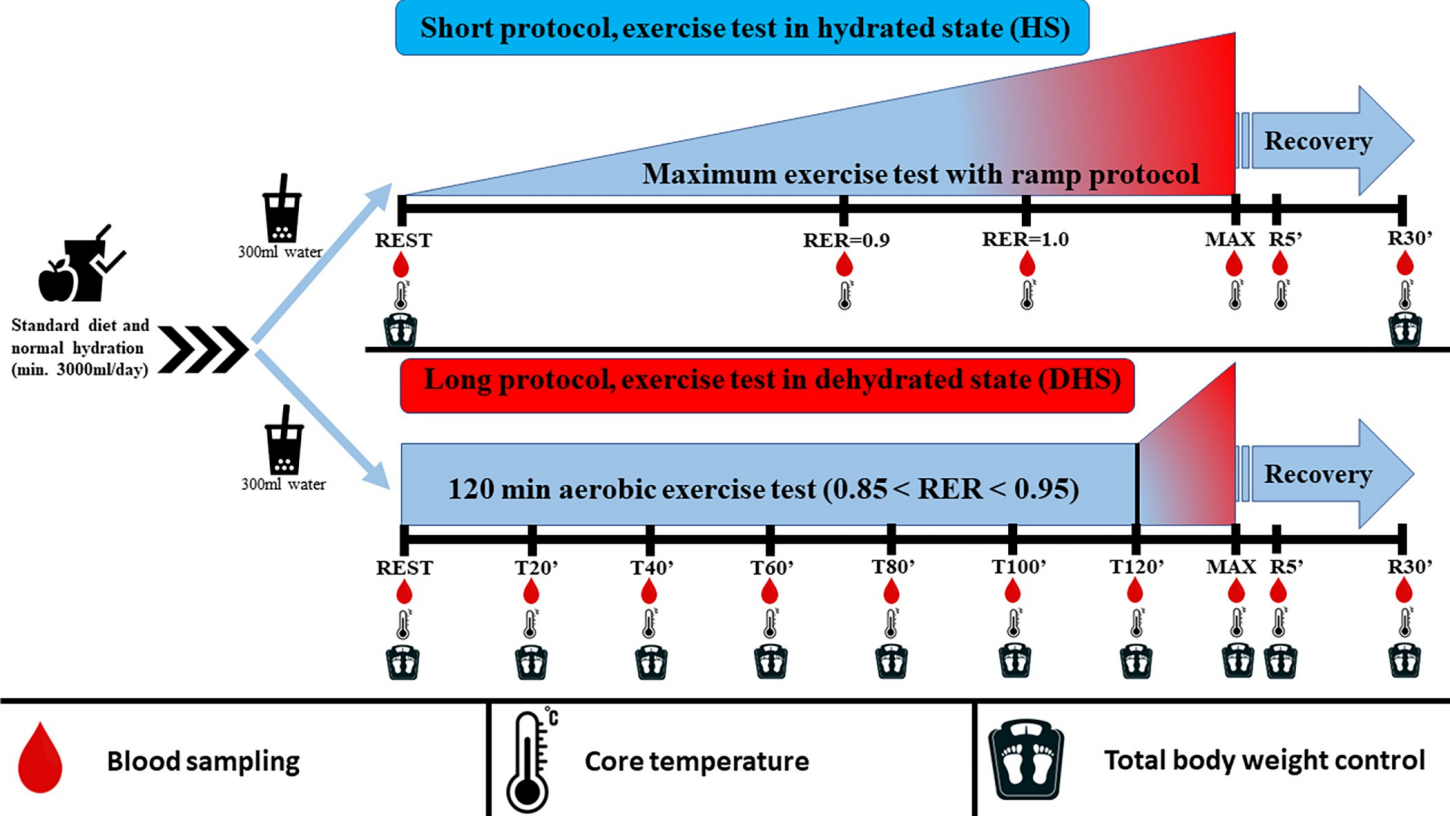
Schematic diagram of exercise protocols and sampling times.

On the second test day (long protocol, dehydrated state, DHS), one week later, an aerobic 120-minute preload was performed on a Lode bicycle ergometer to achieve a dehydrated state at a constant pedal speed of 70-80/min individually setting the intensity with respiratory exchange ratio (RER) between 0.85 and 0.95. The exercise was interrupted every 20 minutes for sampling. The sampling break lasted for 1 min while blood sampling, weight, and body temperature measurements were taken. After 120 minutes, an incremental test was performed, starting from their previous 120-minute individual load intensity, a constant pedal speed of 70-80/min with a 50Watt power increase every 3 minutes until maximum volitional exhaustion. Samples and data were collected at rest before exercise; at times T20’, T40’, T60’, T80’, T100’, T120’ (indicating the 20^th^, 40^th^ … 120^th^ minutes of the exercise), at the maximum load and at the 5^th^ and 30^th^ minutes of the recovery period. Fig 1 summarizes the timing and samplings of our protocol.

During both workloads, a Jaeger Vyntus spirometer was used to monitor VO_2_ during exercise and throughout the recovery period, 30 minutes for the short and 10 minutes for the long protocol. Breath by breath VO_2_, VCO_2_, Respiratory Exchange Ratio (RER), ventilation, and beat-to-beat heart rate were recorded continuously.

Sample collection included heart rate, non-invasive blood pressure (METRONIK BL6, upper left arm cuff), core temperature measured on the eardrum (Braun Thermoscan 5 IRT 4520 ear thermometer), and blood samples (from a forearm intravenous lockable cannula). Bodyweight was controlled before exercise and at the end of the recovery period (ACCUNIQ BC720) in underwear. During the long protocol, we measured body weight at rest with and without sensors, thus, during the 20-minute measurements, the corrected body weight was corrected by subtraction.

Blood samples were taken to clot activator (serum) and EDTA-containing tubes using the Vacuette technique. Blood counts were analyzed from EDTA-containing tubes in accredited laboratory with automated hematology analyzer (Sysmex XN9100). Renal and hepatic function parameters, total protein, and albumin were measured in laboratory conditions from the serum tubes after 15 minutes 3000G centrifugation. In parallel, blood samples were also taken from the intravenous cannula into the heparinized capillary at the specified time points. Hemoglobin, hematocrit, ions (Na^+^, K^+^, Ca^2+^, Cl^-^, anion gap), blood gases (pO_2_, pCO_2_, O_2_-saturation), acid-base parameters (pH, lactate, bicarbonate, base excess), and osmolality were measured (Radiometer ABL 800 Flex). We only reported blood indices that have significant correlation to hemoconcentration and hemodilution, including hemoglobin, hematocrit (Radiometer ABL 800 Flex), serum osmolality, glucose, and lactate.

Some articles suggest the correction of the measured parameters for hematocrit (formula: $\mathrm{calculated}\;\mathrm{parameter}\;\mathrm{value}=\frac{\mathrm{measured}\;\mathrm{parameter}\;\mathrm{value}\;x\;0.50}{\mathrm{measured}\;\mathrm{hematocrit}\;\mathrm{value}}$) due to the phenomenon of plasma shift, the exercise-induced hematocrit increase [21, 22]. This correction is essential for the analysis of certain variables. In the present article, we used uncorrected values, as this is the exercise-induced plasma shift phenomenon and the hemodilution following hemoconcentration is the main focus of our observation.

### Statistical analysis

Our calculations were performed in the Python programming language (version 3.7.3) using the SciPy (1.5.0) package for the statistical tests, Numpy (1.19.1) for the numerical computations, Pandas (1.1.3) for data transformation, and Plotly (5.3.1) for visualization.

Our data were not normally distributed, and the variances did not match, therefore Mann-Whitney U test was used for testing our hypothesis. The threshold of significance was p<0.05. When reporting the results, the median values were given with the first and third quartiles.

Two significance tests were performed at each highlighted measurement time point: we examined the difference between the two exercise protocols (Rest, Max, R5’ and R30’), the change of the parameters within the exercises compared to the resting value.

## Results

Twelve kayak-canoe athletes were tested two-times in a week. Their median body mass index before the short protocol (hydrated state, HS) was 23.34 (q1 22.00; q3 24.13) kg/m^2^ vs. before the long protocol (dehydrated state, DHS) was 23.18 (q1 22.23; q3 24.58) kg/m^2^; p = 0.49 (non-significant; n.s.). 11 athletes out of the 12 completed both exercises, 1 athlete collapsed during the long protocol after 60 min, so his study was interrupted. No injury or other damage occurred during fainting.

### Hemoglobin and hematocrit

At rest, before exercise there was no significant difference in hemoglobin (Hgb) and hematocrit (Hct) values between the short (HS) and long protocol (DHS) days [Hgb median before short protocol 15.6 (q1 15.15; q3 16.1) g/dl vs. Hgb before long protocol median 15.65 (q1 15.43; q3 16.05) g/dl; p = 0.43 (n.s.); Hct median before short protocol 47.9 (q1 46.5; q3 49.35) % vs. Hct median before long protocol 47.85 (q1 47.23; q3 49.25) %; p = 0.42, n.s.].

**Fig 2:**
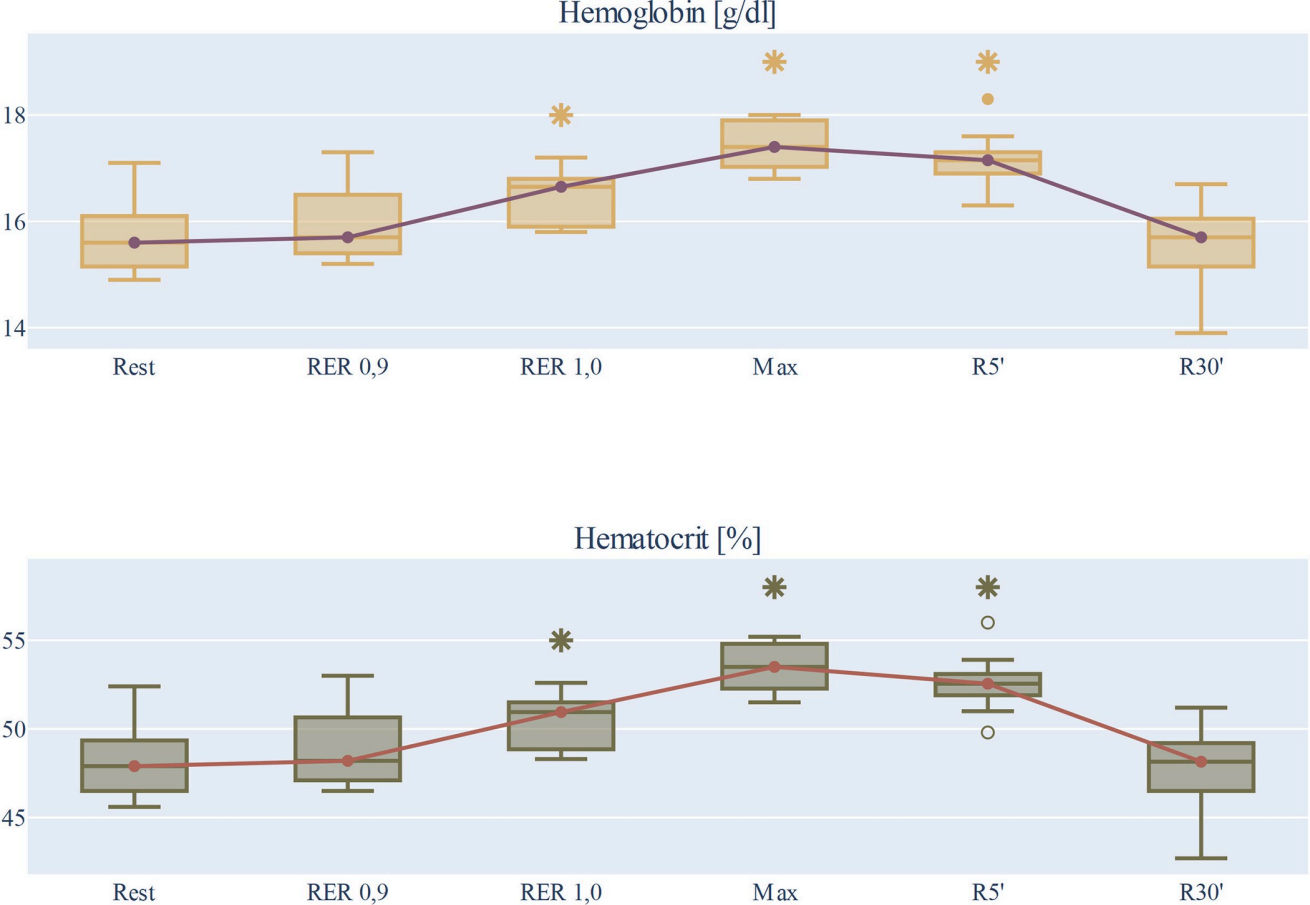
Changes in hemoglobin and hematocrit parameters during the short protocol. Rest: baseline values, sampling time before exercise. RER 0.9: median value 0.9 of the Respiratory Exchange Ratio (the aerobic range of the exercise). RER 1.0: median value 1.0 of the Respiratory Exchange Ratio (the anaerobic threshold of the exercise). Max: sampling time at the maximum exercise. R5’ and R30’: sampling times at the 5^th^ and 30^th^ minutes of the recovery period, respectively. The median, q1 and q3 quartiles, as well as the minimum and maximum values, are represented on the box plots. * Significant differences (p<0.05) from resting values.

During short protocol (HS) exercise, hemoglobin levels and hematocrit increased as follows: in the aerobic range (at value RER = 0.9), the increase in Hgb and Hct was not significant, and then at the anaerobic threshold (at value RER = 1.0) and at the maximum of the load, a pronounced increase in hemoglobin concentration was measured. The highest values of hemoconcentration were measured at the maximum of the load, HS Hgb_Max_ median 17.4 (q1 17.03; q3 17.9) g/dl (p<0.01 compared to resting value); HS Hct_Max_ 53.50 (q1 52.28; q3 54.8) % (p<0.01 compared to resting value). After exercise, we observed a modest decrease in hemoglobin-hematocrit values from the 5^th^ minute of the recovery period, but still significantly higher values were confirmed than the resting parameters (Hgb_R5’_ p = 0.0018 and Hct_R5’_ p = 0.0021 compared to resting value). At the 30^th^ minute of the recovery period, the hemoglobin and hematocrit levels returned to baseline values [Hgb_R30’_ 15.7 (q1 15.15; q3 16.05) g/dl (p = 0.42 n.s.), Hct_R30’_ 48.15 (q1 46.5; q3 49.2) % (p = 0.43 n.s.)]. In the normal hydrated state, during the short protocol, the hemoglobin and hematocrit values are demonstrated in Fig 2.

The long protocol (DHS) exercise was interrupted every 20 min for sampling and to control the degree of dehydration (with measuring body weight loss). As demonstrated in Table 1, hemoglobin-hematocrit values measured every 20 min increased discreetly (significant increase from the 80^th^ minute, p = 0.019), and then after the 120 minutes preload, an even more significant increase of hemoglobin-hematocrit levels were detected at maximum performance (p = 0.005, compared to resting value). At the 5^th^ minute of the recovery period, the hemoconcentration decreased and then after 30 minutes it was diluted to the resting, pre-load (hydrated) state.

**Table 1:** Hemoglobin and hematocrit values during the long, dehydration protocol.

	Hemoglobin (g/dl)			Hematocrit (%)		
Timing	median	(q1; q3)	p- value*	median	(q1; q3)	p- value*
**Rest**	**15.65**	(15.43; 16.05)		**47.85**	(47.23; 49.25)	
**T20’**	**16.10**	(15.80; 16.60)	0.070	**49.40**	(48.48; 50.85)	0.070
**T40’**	**16.25**	(16.03; 16.33)	**0.028**	**49.80**	(49.25; 50.13)	**0.028**
**T60’**	**16.35**	(15.95; 16.63)	0.053	**50.05**	(48.88; 51.03)	0.056
**T80’**	**16.50**	(15.95; 16.85)	**0.019**	**50.40**	(48.95; 51.55)	**0.018**
**T100’**	**16.30**	(16.10; 16.70)	**0.045**	**49.90**	(49.30; 51.30)	**0.042**
**T120’**	**16.50**	(15.85; 16.80)	**0.037**	**50.70**	(48.55; 51.55)	**0.035**
**Max**	**16.90**	(16.43; 17.60)	**0.005**	**51.90**	(50.35; 53.93)	**0.005**
**R5’**	**16.20**	(15.75; 16.68)	0.054	**49.80**	(48.30; 51.10)	**0.045**
**R30’**	**15.75**	(15.48; 16.13)	0.454	**48.25**	(47.48; 49.45)	0.386

Rest: baseline values, sampling time before exercise; T20’, T40’,…, T120’: sampling times at 20^th^, 40^th^, …, 120^th^ minutes of the exercise, respectively. Max: sampling time at the maximum power after 120 min preload; R5’ and R30’: sampling times at the 5^th^ and 30^th^ minutes of the recovery period, respectively.

* p- values were calculated using the U test model. Significant differences from resting values are highlighted in bold in the p-value columns.

Compared to the short protocol, the degree of hemoconcentration at the maximum load did not show a significant difference and there was no difference between the two groups in the recovery period (Hgb_Max_ p = 0.40; Hct_Max_ p = 0.06; Hgb_R5’_ p = 0.06; Hct_R5’_ p = 0.03; Hgb_R30’_ p = 0.42 n.s.; Hct_R30’_ p = 0.41, n.s.).

### Bodyweight

At rest before the short protocol, subjects weighed an average of 75.5 (q1 73.95; q3 79.93) kg, body water measured by body impedance analysis (BIA) was 50.15 (q1 48.55; q3 52.73) %, and fat-free mass (FFM) was 68.60 (q1 66.30; q3 72.0) kg. No fluid or energy intake was allowed until the end of the protocol. The median body weight of the athletes was 74.3 (q1 72.88; q3 79.0) kg after exercise, corresponding to total body mass (TBM) loss of 1.18% (q1 1.05; q3 1.50; p = 0.23 n.s.).

Prior to the long protocol, subjects weighed 75.9 (q1 74.0; q3 77.0) kg, according to BIA with 50.0 (q1 48.7; q3 52.58) % body water and 68.45 (q1 66.0; q3 70.33) kg FFM. These baseline values were the same as that of the short protocol (p > 0.05 respectively). During dehydration, weight loss was measured in every 20^th^ min. Weight loss was defined as total body mass (TBM), weight loss, and percentage of TBM loss. Body weight records during dehydration are summarized in Table 2. At the end of the procedure, subjects achieved a 3.69 (q1 3.23; q3 3.90) % TBM loss, corresponding to a fluid loss of 2.80 (q1 2.40; q3 3.1) liters.

**Table 2:** Changes in bodyweight, rate of weight loss (kg) and % of the total body mass during the long dehydration protocol.

	Bodyweight loss (kg)			Percentage of TBM (%)			Bodyweight (kg)		
Timing	median	(q1; q3)	p- value*	median	(q1; q3)	p- value*	median	(q1; q3)	p- value*
**Rest**	**0.00**			**0.00**			**75.90**	(74.0; 77.0)	
**T20’**	**0.20**	(0.0; 0.35)	**<0.001**	**0.26**	(0.0; 0.43)	**<0.001**	**75.50**	(73.85; 76.9)	0.420
**T40’**	**0.50**	(0.40; 0.55)	**<0.001**	**0.66**	(0.54; 0.71)	**<0.001**	**75.20**	(73.4; 76.55)	0.343
**T60’**	**0.80**	(0.70; 0.90)	**<0.001**	**1.05**	(0.95; 1.17)	**<0.001**	**75.00**	(73.2; 76.0)	0.194
**T80’**	**1.10**	(0.9; 1.15)	**<0.001**	**1.45**	(1.22; 1.49)	**<0.001**	**75.00**	(73.1; 75.6)	0.148
**T100’**	**1.40**	(1.37; 1.50)	**<0.001**	**1.84**	(1.77; 1.95)	**<0.001**	**74.50**	(72.6; 75.45)	0.115
**T120’**	**1.70**	(1.58; 1.75)	**<0.001**	**2.24**	(2.13; 2.27)	**<0.001**	**74.10**	(72.3; 75.35)	0.088
**Max**	**1.70**	(1.70; 1.88)	**<0.001**	**2.27**	(2.15; 2.44)	**<0.001**	**73.90**	(72.25; 75.05)	0.083
**R5’**	**1.70**	(1.70; 1.90)	**<0.001**	**2.39**	(2.22; 2.59)	**<0.001**	**73.90**	(72.25; 75.0)	0.074
**R30’**	**2.80**	(2.40; 3.10)	**<0.001**	**3.69**	(3.23; 3.90)	**<0.001**	**72.60**	(71.4; 74.4)	**0.043**

Rest: baseline values, sampling time before exercise; T20’, T40’, …, T120’: sampling times at 20^th^, 40^th^, …, 120^th^ minutes of the exercise, respectively. Max: sampling time at the maximum power after 120 min preload; R5’ and R30’: sampling times at the 5^th^ and 30^th^ minutes of the recovery period, respectively. TBM: total body mass

* p- values were calculated using the U test model. Significant differences from resting values are highlighted in bold in the p-value columns.

### Glucose and lactate

During the HS protocol, the resting blood glucose level was 4.85 (q1 4.68; q3 5.05) mmol/l, followed by an increase at the anaerobic threshold and at the maximum load, which became even more pronounced at the 5^th^ minute of the recovery, then decreased values were observed in the 30^th^ minute of the rest. Blood glucose and lactate concentration during the HS protocol are summarized on Table 3.

**Table 3:** Blood glucose and lactate concentration during the short protocol.

	Serum glucose (mmol/l)			Serum lactate (mmol/l)			Heart rate (beat/min)		
Timing	median	(q1; q3)	p- value*	median	(q1; q3)	p- value*	median	(q1; q3)	p- value*
**Rest**	**4.85**	(4.68; 5.05)		**0.9**	(0.70; 1.13)		**64.5**	(60; 73.75)	
**RER 0.9**	**4.95**	(4.60; 5.30)	0.397	**1.75**	(1.45; 2.35)	**<0.001**	**142**	(120.75; 146)	**<0.001**
**RER 1.0**	**5.40**	(5.13; 5.58)	**0.018**	**4.45**	(3.23; 5.90)	**<0.001**	**159**	(148; 168.25)	**<0.001**
**Max**	**5.70**	(5.35; 5.90)	**0.006**	**13.0**	(12.2; 14.95)	**<0.001**	**187**	(180.75; 195.25)	**<0.001**
**R5’**	**7.10**	(6.45; 7.45)	**<0.001**	**16.0**	(13.75; 17.0)	**<0.001**	**116**	(105.5; 119)	**<0.001**
**R30’**	**5.65**	(5.30; 5.95)	**0.019**	**6.45**	(5.43; 8.65)	**<0.001**	**94.5**	(86.5; 100)	**<0.001**

Rest: baseline values, sampling time before exercise. RER 0.9: median value 0.9 of the Respiratory Exchange Ratio (the aerobic range of the exercise). RER 1.0: median value 1.0 of the Respiratory Exchange Ratio (the anaerobic threshold of the exercise). Max: sampling time at the maximum exercise. R5’ and R30’: sampling times at the 5^th^ and 30^th^ minutes of the recovery period, respectively.

* p- values were calculated using the U test model. Significant differences from resting values are highlighted in bold in the p-value columns.

During the dehydration (DHS) protocol, we sought to ensure that normal blood glucose and lactate levels during exercise so that neither substrate availability nor acid-base shift affected performance. Blood glucose was between 4 and 5 mmol/l and lactate was below 2 mmol/l during the 120-minute preload (Table 4).

**Table 4:** Blood glucose and lactate values during the long protocol.

	Serum glucose mmol/l			Serum lactate mmol/l		
Timing	median	(q1; q3)	p- value*	median	(q1; q3)	p- value*
**Rest**	**5.10**	(4.58; 5.23)		**0.95**	(0.90; 1.08)	
**T20’**	**4.70**	(4.50; 4.88)	0.052	**1.95**	(1.78; 2.73)	**<0.001**
**T40’**	**4.80**	(4.70; 4.90)	0.086	**1.60**	(1.45; 1.85)	**<0.001**
**T60’**	**4.75**	(4.50; 5.13)	0.141	**1.50**	(1.40; 1.73)	**<0.001**
**T80’**	**4.80**	(4.50; 4.95)	**0.046**	**1.40**	(1.25; 1.55)	**<0.001**
**T100’**	**4.70**	(4.40; 5.20)	0.126	**1.60**	(1.45; 1.70)	**<0.001**
**T120’**	**4.90**	(4.50; 5.20)	0.229	**1.80**	(1.45; 1.95)	**<0.001**
**MAX**	**4.75**	(4.00; 4.98)	0.099	**6.65**	(4.80; 7.90)	**<0.001**
**R5’**	**6.10**	(4.85; 6.65)	0.065	**6.40**	(4.30; 6.80)	**<0.001**
**R30’**	**4.85**	(4.60; 5.43)	0.465	**1.65**	(1.28; 2.43)	**<0.001**

Rest: baseline values, sampling time before exercise; T20’, T40’, …, T120’: sampling times at 20^th^, 40^th^, …, 120^th^ minutes of the exercise, respectively. Max: sampling time at the maximum power after 120 min preload; R5’ and R30’: sampling times at the 5^th^ and 30^th^ minutes of the recovery period, respectively.

* p- values were calculated using the U test model. Significant differences from resting values are highlighted in bold in the p-value columns.

### Performance

During the short protocol (HS), subjects achieved a maximum power of 301.5W (q1 290.5; q3 312.8), VO_2max_ was 3932 ml/min (q1 3675; q3 4028), relative VO_2max_ 50.1 ml/min/kg (q1 48.0; q3 52.4) at the end of the gradually increasing intensity. During the recovery period, VO_2_ and HR values decreased [VO_2R5’_ 690.5 ml/min (q1 667.75; q3 731.25); HR_R5’_ 115.5/min (q1 102.75; q3 118.75)], after 30 minutes, the VO_2_ returned to baseline values, however, the heart rate were still elevated [VO_2R30’_ 525.5 ml/min (q1 430.8; q3 559.05), p = 0.52; HR_R30’_ 92/min (q1 89.5; q3 97.5) p<0.01].

During the long protocol, the first 120 minutes were performed at a constant power, averaging 125W (q1 125; q3 150). During the 120 min, the load intensity was determined individually based on the RER (0.85–0.95), ensuring that sufficient intensity was achieved for all participants, while staying in the aerobic range. The average RER, VO_2_, and heart rate values per 20-minute cycle were summarized in Table 5. After 120 minutes, a gradually increasing workload was applied until maximal volitional exertion. The maximum power of long protocol was 240W (q1 210; q3 247.5).

**Table 5:** Respiratory exchange ratio, VO_2_ and heart rate values during the long dehydration protocol.

	RER		VO2 (ml/min)		Heart rate (beat/min)		
Timing	median	(q1; q3)	median	(q1; q3)	median	(q1; q3)	p- value*
**Rest**	**0.78**	(0.74; 0.8)	**492.5**	(458; 624.8)	**79.5**	(77.8; 85.0)	
**T20’**	**0.91**	(0.90; 0.94)	**1979.0**	(1909.8; 2067.8)	**123.5**	(119.5; 132.8)	**<0.001**
**T40’**	**0.89**	(0.88; 0.92)	**2183.5**	(1922.3; 2249)	**136.0**	(131; 149.3)	**<0.001**
**T60’**	**0.86**	(0.85; 0.90)	**2249.5**	(2112.5; 2300.5)	**142.5**	(134; 151.8)	**<0.001**
**T80’**	**0.88**	(0.83; 0.93)	**2269.0**	(2046.8; 2326.3)	**150.0**	(141.5; 160.5)	**<0.001**
**T100’**	**0.86**	(0.84; 0.87)	**2268.5**	(2123.5; 2365.5)	**157.5**	(146.8; 163.3)	**<0.001**
**T120’**	**0.85**	(0.83; 0.86)	**2291.0**	(2137.3; 2448.8)	**164.5**	(149.5; 171)	**<0.001**
**Max**	**1.03**	(0.99; 1.11)	**2555.0**	(2491.5; 2688)	**171.0**	(158.5; 179.5)	**<0.001**
**R5’**	**0.89**	(0.86; 0.91)	**1136.0**	(1051.3; 1175.5)	**144.5**	(128.8; 153.8)	**<0.001**
**R30’**	**none**	none	**none**	none	**97.0**	(88.8; 101.8)	**<0.001**

Rest: baseline values, sampling time before exercise; T20’, T40’, …, T120’: sampling times at 20^th^, 40^th^, …, 120^th^ minutes of the exercise, respectively. Max: sampling time at the maximum power after 120 min preload; R5’ and R30’: sampling times at the 5^th^ and 30^th^ minutes of the recovery period, respectively. RER: Respiratory exchange ratio

* p- values were calculated using the U test model. Significant differences from resting values are highlighted in bold in the p-value columns.

**Fig 3:**
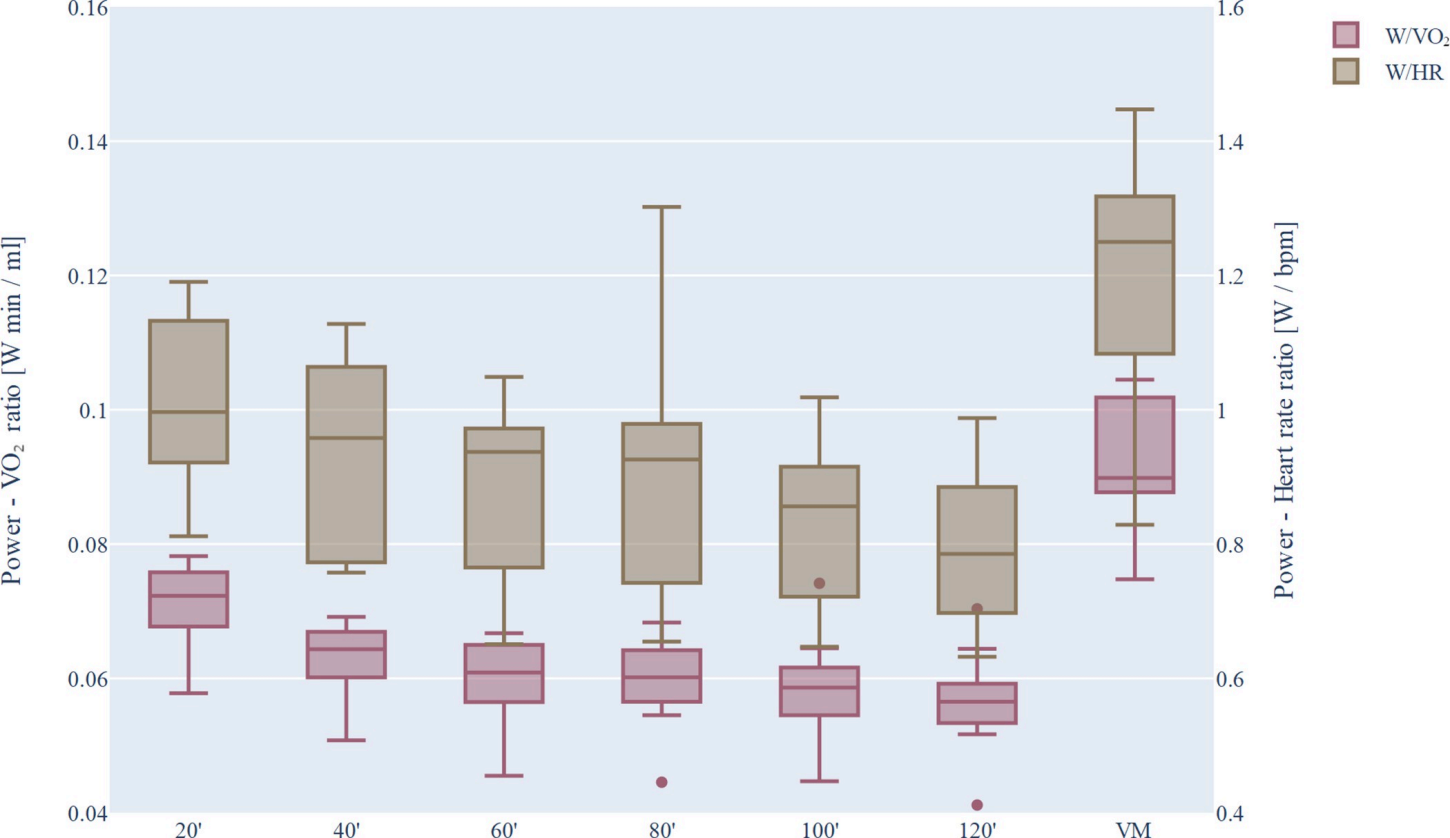
Power/VO_2_ and power/heart rate ratios as a function of time during the long dehydration protocol. 20’, 40’, …, 120’: averages of VO_2_ and heart rate parameters measured over 20-minute workout periods, respectively. VM: sampling time at the maximum power after 120 min preload. The median, q1 and q3 quartiles, as well as the minimum and maximum values, are represented on the box plots.

Although the power output was constant during the 120-min preload, the same RER, glucose, and lactate values were recorded, but VO_2_ and heart rate increased steadily. The W/VO_2_ and W/HR ratios decrease during exercise, ie. the same performance can be maintained only at higher circulation and respiration parameters. The continuous deterioration in cardiopulmonary efficiency were represented in Fig 3.

### Core temperature

In the short protocol, the core temperature increased significantly at the maximum of the load (HS resting core temperature median 36.8°C (q1 36.5; q3 36.95), Max 37.4°C (q1 37.25; 37.9) p = 0.0045), and then a further increase was measured in the 5^th^ minute of the recovery period (R5’ median 37.8°C (q1 37.45; q3 37.85), p = 0.0031 compared to resting values). Thirty minutes after exercise, it returned to resting levels (R30’ median 36.7°C (q1 36.55; q3 37.0), p = 0.77).

During the first 120 minutes of the long protocol, the resting temperature (36.6 (q1 36.4; q3 36.83)°C rose continuously till the maximum load, reaching the highest temperature (38.2 (q1 37.4; q3 38.35)°C; p = 0.0003). A decreasing trend was observed during the recovery period, however, after 30 minutes the core temperature was significantly higher than the resting value (median 37.0 (q1 36.76; q3 37.35)°C, p = 0.003).

### Osmolality

Serum osmolality increased during both exercises. In HS protocol, from the baseline 288.5 (q1 287.8; q3 292.1) mOsm value, the increase in osmolality at the anaerobic threshold (RER = 1.0 and above) became significant, reaching its maximum at the peak of the workload (median 304.4 (q1 303.3, q3 308) mOsm/l, p = 0.001 compared to resting value), then started to return to baseline at 30^th^ min of restitution (R5’ 298.5 (q1 292.6; q3 298.6) mOsm, p = 0.01; R30’ 289.6 (q1 287.5; q3 291.5) mOsm, p = 0.49). During the DHS protocol, from the resting 291 (q1 289.8; q3 293.9) mOsm, the osmolality increased gradually, reaching its maximum value at the peak of the load (306.25 (q1 304.25; q3 313.3) mOsm, p<0.001). Osmolality started to return to baseline during recovery but stayed significantly elevated (298.15 (q1 296.65; q3 301.73) mOsm, p<0.001) at the 30^th^ minute of the rest. There was no significant difference between the maximum serum osmolality values measured during the HS and DHS protocol (p = 0.49). At time R30’, there was a significant difference in osmolality between the two exercises (p<0.001).

**Fig 4:**
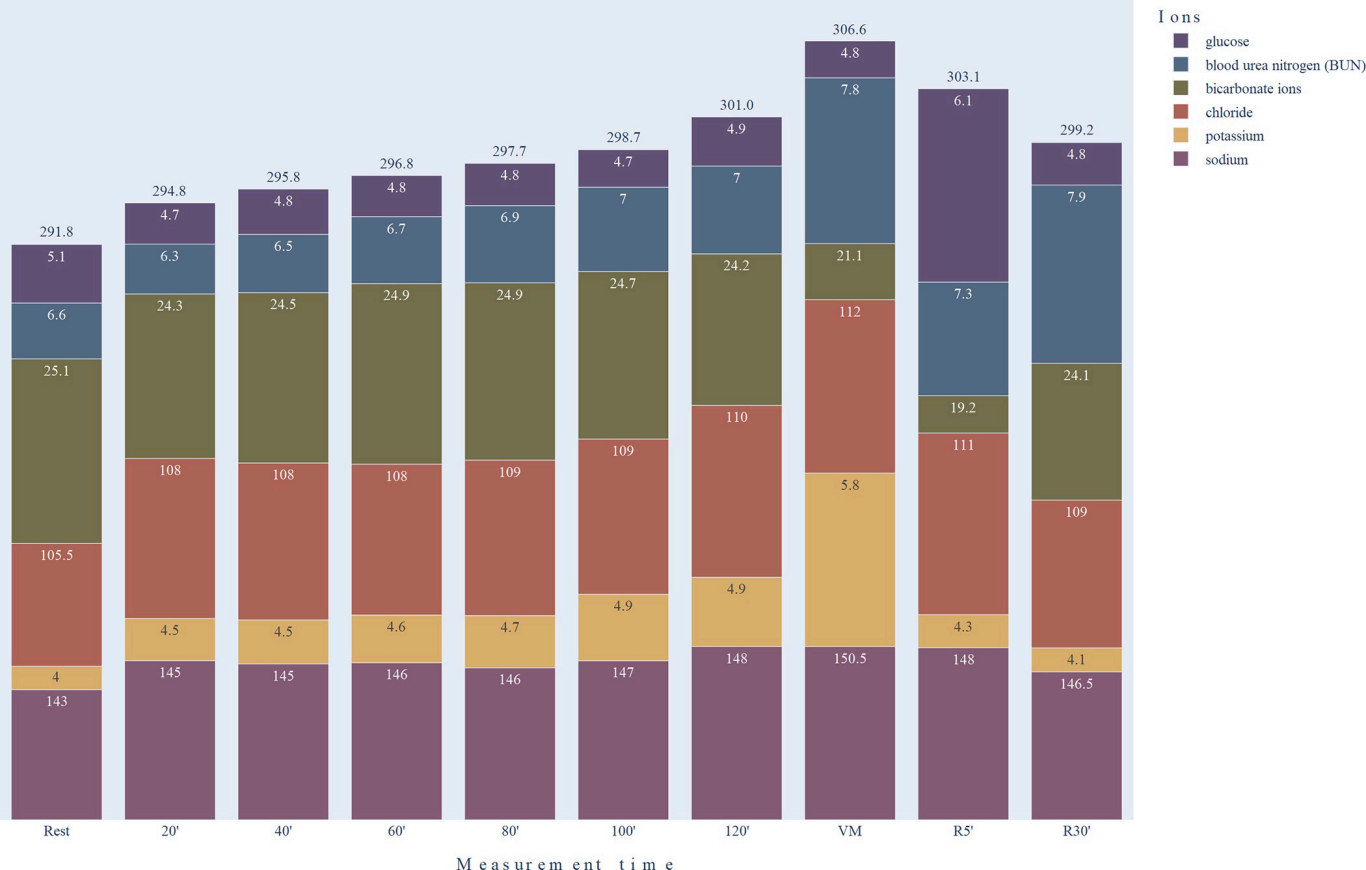
Changes in major blood components influencing plasma osmolality during the long dehydration protocol. Rest: baseline values, sampling time before exercise; 20’, 40’, …, 120’: sampling times at 20^th^, 40^th^, …, 120^th^ minutes of the exercise, respectively. VM: sampling time at the maximum power after 120 min preload; R5’ and R30’: sampling times at the 5^th^ and 30^th^ minutes of the recovery period, respectively. A major part of the osmolality is given by the change of sodium ions. To better represent visually the changes in the other components, we used an exponential magnification of the values, however, the numbers on the graphs show the actual values. The height of the columns and the numbers at the top indicate the changes in osmolality values.

Blood osmolality is predominantly determined by sodium, potassium, chloride and bicarbonate ions, blood urea nitrogen, and glucose, which may play a more significant role during exercise due to effluent catecholamines. Besides, plasma proteins, primarily albumin fraction, also play an essential role in regulating plasma colloid-oncotic pressure. The changes of the main variables as a function of time are represented in Fig 4.

## Discussion

### Hemoconcentration and hemodilution

In our study, 12 elite kayak-canoe athletes were examined with two different protocols in a short period of time to provide the same training levels between the two test exercises. Subjects arrived with the same body composition and hydration levels before the two protocols. At first, a maximum step protocol was performed in the normal hydrated state, then a dehydrated state was modeled with a 120-min preload, followed by an incremental maximum workload test. We focused on a more accurate understanding of the phenomenon of hemoconcentration, and post-exercise hemodilution, and the changes of the blood components.

During intense physical activity, the blood thickens and hemoconcentration develops, which is confirmed by the current and previous results of our working group. On the course of the last decades, several research groups have reported that intense muscle metabolism, increased sympathetic autonomic nervous system activity, systolic blood pressure, and high intensity exercise result in a mechanism of plasma shift [2–4, 16, 17], which causes hemoconcentration within the intravascular system. Anaerobic spleen contraction, which has been intensively researched in recent years by Shepard, Schagatay et al., may also contribute to this [5, 23, 24]. Exercise related hemoconcentration may exert short term benefits, increase performance, and the maximum cardiac output can provide more efficient muscle oxygenation and substrate supply by circulating concentrated blood. During exercise, the elevated density of the blood is compensated by the high flow rate and raised core temperature [25]. According to Sawka, hypohydration does not impair submaximal intensity aerobic performance in a cold environment, however, in heat it impairs efficiency in direct proportion to increasing skin circulation [26]. The advantage of hemoconcentration is supported by the results of Holland et al., where hydration (i.e., dilution of hemoconcentration) during 1 hour of intense exercise was shown to impair performance [8]. However, it is also a known fact that, during long term exercises, fluid loss and dehydration can be detrimental to performance [14, 26] or even dangerous to health [9–13]. According to Holland et al., fluid replenishment was associated with a 2% increase in performance at 1–2 h on medium-intensity exercise (60–70% of VO_2_max) and 3% at exercise over 2 hours [8].

The exact transition between beneficial and detrimental hemoconcentration is unknown, although knowledge of this would be essential for athletic performance and health. Therefore, further studies are needed for a more accurate understanding.

Under normal hydration (HS), exercise-related hemoconcentration is a short-term process: hemoglobin-hematocrit begins to rise from the anaerobic threshold and reaches its maximum at the peak load. After exercise, hemodilution was observed, reaching the resting value 30 minutes after loading. Our previous study also highlighted the fact that hemodilution may be slower than the recovery of the circulatory system, thus the blood viscosity flow compensation can be temporarily impaired (“Critical Hemoconcentration Zone”) [19].

During the second workload (DHS), physiological dehydration was exerted: subjects were loaded for 120 min, maintaining constant power output in the aerobic range on a cycloergometer. An important consideration in our study design was to achieve dehydration with a sport-relevant method, with exercise, without diuretics or external heat effects, e.g. with sauna. The low intensity of the exercise is evidenced by the fact that lactate values stayed below 2 mmol/l and glucose concentration, measured in every 20 minutes, remained in the normal physiological range. Without fluid replacement, body weight of subjects decreased continuously, achieving an average TBM loss of 3.69% by the end of the load. During the 120 min preload, the hemoconcentration increased minimally till the first 20 min and then kept on increasing discretely despite the continuous fluid loss. As the intensity increased (after 120 min), additional significant hemoconcentration occurred. Without rehydration, hemodilution occurred after exercise, similar to the HS protocol, hemoglobin and hematocrit decreased back to resting values within 30 min.

Maughan et al. examined marathon runners before and immediately after the race, within 2–4 minutes. Significant plasma loss has been described [27]. According to our study, post-exercise hemoglobin and hematocrit values do not correlate with the degree of fluid loss, which can be explained by the dynamic hemoconcentration due to physical intensity. We found that the phenomenon of hemoconcentration up to the average 3.69% TBM loss is not significantly influenced by the extent of hydration, but the intensity of the workload, which can be related to the phenomenon of plasma shift and spleen contraction. Serum osmolality is a more accurate marker of dehydration than the blood hemoglobin or hematocrit values. In the hydrated state (HS), similar to the hemoconcentration, the osmolality increased significantly and then returned to the resting level within 30 minutes. In the dehydrated state (DHS), osmolality also increased during exercise, however, 30 minutes after exercise it was still significantly higher than the baseline value.

Dehydration induced by physical exercise leads to hypertonic hypovolemia due to plasma water loss [22, 28]. Blood biomarkers of hemoconcentration, such as blood osmolality and sodium levels, are therefore widely used as an index of dehydration. According to the literature, blood osmolality is a more sensitive marker compared to serum sodium, so it can be used to monitor the level of hydration in athletes [29–31]. Recently, experiments have been performed on the analysis of salivary osmolality, salivary flow rate, sweat, and tears. These methods may represent useful improvements in the measurement of hydration status in sports since sampling is non-invasive; their sensitivity and specificity have yet to be clarified [29, 31].

During long-term exercise without fluid and carbohydrate supplementation, the glycogen content of the muscles is depleted, thereby reducing the water-binding capacity of the muscles [32]. This process would facilitate osmotic dilution of intravascular concentrated blood after exercise; however, it is limited by the fluid deficiency, according to our results. Recent data suggest that glycogen depletion alone does not alter the body water distribution in the intra- and extracellular spaces [33].

The sites of fluid loss during dehydration have not been sufficiently clarified. Several working groups reported that dehydration impairs cognitive function, visual and short-term memory, enhances fatigue, moreover can cause prolonged reaction time [34–37]. These data may suggest dehydration of the central and peripheral nervous system. However, several working groups have reported that much of the lost fluid is missing from the muscle [38, 39], but further investigation is needed to clarify this. Separate changes in hematocrit and osmolality after exercise draw attention to the fact that spleen relaxation may play a greater role in hemodilution, which removes erythrocytes concentrate from the blood, thus not being affected by osmolality. Further studies are planned in this direction.

One more plausible, but speculative explanation could be for the mechanism of plasma shift is the mechanically activated Piezo system. Piezo ion channels are the largest transmembrane protein family encoded by two genes in humans, namely Piezo1 and Piezo2. They have an important, possibly fundamental role in homeostasis maintenance and mechanotransduction in various tissues [40–46], especially responding to stretch and shear stress [47, 48]. Noteworthy, that Piezo1 ion channels have a role in cell alignment [41, 45], regulation of osmolality and aqueous humor outflow dynamics in other tissues [49, 50], not to mention shear stress detection [41, 45]. In the contrary, Piezo2 ion channels maintain homeostasis largely in somatosensory neurons and provide the principal mechanotransduction channel for proprioception [41, 42, 44, 51]. Correspondingly, increased hemoconcentration could elevate shear stress detection through Piezo1 of endothelial cells. Furthermore, excited endothelial cells, as neuromodulators, could stimulate Piezo2 of somatosensory terminals in the resistance arteries, leading to their vascular dilatation. Moreover, endothelial Piezo1 could regulate osmolality and water outflow dynamics in correlation with exercise intensity and especially fatigue induced somatosensory hyperexcitability. Indeed, there is evidence, that osmotic derived erythrocyte swelling increases the stretch activation of Piezo1 in order to counter act the water influx [49, 52].

Not to mention, that Piezo1 of striated muscle membranes could contribute in an analogous way, but more actively to the aforementioned enormous muscle fluid loss. The finding is suggestive of this proposed mechanism machinery that Piezo1 channels sense and respond not only in spatially-restricted manner [52], but the entire body holistically to enhance performance and reset cardiovascular homeostasis accordingly [53].

### Bodyweight and performance

When using changes in body mass, it is assumed that the acute TBM loss of 1g is equivalent to the loss of 1mL of water. There is evidence in the literature that 2% of total weight loss can be detrimental to performance [9, 10, 14]. Our study represents that the subjects were able to maintain constant power output even during the first 20–40 minutes with increasing heart rate and VO_2_ values, although the rate of weight loss was only 0.26–0.66% of TBM. Fatigue is obviously a complex process, but with a normal supply of nutrients and acid-base homeostasis, muscle efficiency has continuously deteriorated in our observation. A review article by Cheuvron and Kenefick also emphasizes that the performance-impairing effects of 2% TBM loss may have negative effects of multiple sports skills. The effect of dehydration on a particular sporting skill or task is likely to depend on the structure of the task itself (e.g., endurance, strength, cognitive, and motor skills) [54]. For this reason, several sport-specific data would be needed on hydrations that are optimal, maintain or improve performance under specific environmental conditions (temperature, relative humidity, altitude, etc.). By refining the data, we can get closer to understanding the positive and negative hemoconcentration and thus properly hydrate our athletes at the optimal time, avoiding health damage and achieving better sports performance. Our results can contribute to optimizing the training program of not only elite sports but also recreational athletes.

## Conclusion

Both physical exercise and dehydration can alter the distribution and amount of liquid between fluid spaces. In our study, the effect of acute euhydrated and a long-term dehydrated workload were examined on the phenomenon of hemoconcentration and post-exercise hemodilution. Hemoconcentration is essentially an exercise-related phenomenon, and previous, significant dehydration of 3.69% did not increase the degree of hemoconcentration as expected and had no effect on the dynamics of post-exercise hemodilution, although similar hemoconcentration was seen at lower peak load (Watt) in dehydrated state. In both hydrated and dehydrated states, hemoconcentration started to develop under aerobic extensive loading and reached its peak at maximum exercise. The dynamics of hemodilution was similar in the dehydrated and hydrated states of the athletes. In both cases, a minor extent of hemodilution was observed during the first 5 minutes of the recovery after maximal exercise, and hemoglobin-hematocrit values finally returned to resting values by the 30^th^ minute of the resting period.

### Practical application

Significant higher hemoglobin and hematocrit levels have been reported during exercise, which may potentiate the performance impairment or cause even detrimental effects on health. Neither adequate hydration nor significant (3.69%) dehydration influenced the degree of hemoconcentration, so it can be assumed that the phenomenon cannot be eliminated or affected by proper hydration alone. Following high-intensity sprint sports, maximal performance leads to significant hemoconcentration, after which hemodilution may occur later than cardiovascular relaxation. Therefore, it is crucial to apply a gradual cool-down period after high-intensity workloads so that the dynamics of hemodilution is synchronized with the relaxation of the circulation.

### Study limitations

In the present study, we examined 12 male kayak-canoe athletes. Future research with a more significant number of elements is necessary, which covers female athletes and other sports. The workload of the present study required advanced aerobic endurance, so untrained volunteers could not be selected as a control group.

## Supporting information

S1 Table Database of the short (HS) and long (DHS) protocol. Rest: baseline values, sampling time before exercise. RER 0.9: median value 0.9 of the Respiratory Exchange Ratio (the aerobic range of the exercise). RER 1.0: median value 1.0 of the Respiratory Exchange Ratio (the anaerobic threshold of the exercise). Max: sampling time at the maximum exercise of the short protocol. VM: sampling time at the maximum of the long protocol exercise. 20’, 40’, …, 120’: sampling times at 20^th^, 40^th^, …, 120^th^ minutes of the long protocol exercise, respectively. R5’ and R30’: sampling times at the 5^th^ and 30^th^ minutes of the recovery period, respectively. SMM: skeletal muscle mass; ICW: intracellular water; ECW: extracellular water.
(XLSX)
